# Book Reviews

**Published:** 1800-11

**Authors:** 


### CRITICAL RETROSPECT
OF
### MEDICAL AND PHYSICAL LITERATURE.

### [ FOREIGN AND DOMESTIC. ]

*Recherches sur l'Influence de l'Air dans le Developpement*; i. e. Researches on the Influence of Air in causing Diseases, on their Character and Treatment; a Work, in which it is proposed to establish, on Principles of Medicine and Observation, the Relation of Nosologic Constitutions; by L. D. A. Bouffey, Physician at Argentan. Paris, Year 7, for Didot the younger, pp. 176.

To enquire into the influence of the atmospheric constitution, in producing diseases, is an undertaking both extensive and difficult; and though it is a subject of the greatest importance, it has not yet sufficiently been investigated, and still requires to be ascertained by experience and the evidence of facts. The author of this publication has, therefore, a just claim to the praise of the public, for having devoted himself to this task by collecting materials for the purpose, and arranging them in such a way, that a more perfect theory can be founded upon them. The work, of which the author has published this first volume, is divided into *three parts*. *In the first*, which is comprehended in this volume, the author, after having explained the general principles relating to meteorology, subjoins some remarks on the necessity of observing the vicissitudes of the atmosphere, on the defects to which this sort of observation is still subject, and on the means of rendering it more useful to medicine. He proceeds farther to examine the atmospheric air, by considering it with respect to its physical and chemical properties, and at last in its state of agitation. Under this head, the winds, their origin, and the influence they have on atmospheric constitutions, and on the seasons, ought to be likewise recorded in this general survey, as without these the examination of each particular temperature will be rendered less easy. After having examined the physical causes, it would be necessary to represent under a general view, their action on the animal œconomy; and this is the point to which the author has aimed, by examining the effects of atmospheric air, or rather its decomposition in the lungs, and the impression it occasions in the interior organisation. *In the second part*, the author means to treat particularly of the four principal temperaments, as adopted by *Hippocrates; and in the third part*, he is to consider the air, when charged with heterogeneous particles, in a greater proportion than it generally is found to contain, which he comprehends under four

*four* different claſſes. In the *firſt*, he is to examine the matter of electricity, as far as it influences and alters the atmoſpheric air; in the *ſecond*, the mineral exhalations; in the *third*, the vegetable; and in the *fourth*, the animal exhalations.

---

*Reflexions ſur la Nouvelle Methode d'Inoculer la petite Verole avec le Virus de Vacches*; i. e. Reflexions on the New Method of Inoculating with the Vaccine Poiſon, by T. S. VAUME, M. D. Paris; a pamphlet of 11 leaves.

ACCORDING to the author of this Pamphlet, the inoculation of the ſmall-pox, undertaken by well-informed perſons, leaves nothing farther to be wiſhed, and is quite ſatisfactory. In this reſpect he appeals to the numerous experiments made by himſelf with the greateſt ſucceſs, in his practice at Agacio. He conſiders the fever, and other ſymptoms which precede, accompany, and follow the eruption, as neceſſary requiſites for the ſucceſs of inoculation, obſerving, at the ſame time, that it is always in the power of a phyſician to ſubdue them. Upon theſe premiſes he concludes by aſſerting, that the new method of inoculating with the cow-pox, (vaccine, called by him vacchine) ought to be utterly rejected, even without any farther examination.

---

*Voyage dans l'Empire de Flore;* i. e. Travels in the Empire of Flora; or, Elements of Vegetable Natural Hiſtory; by L. M. P. T. Phyſician, 2 vol. 8vo. Paris, Méquignon.

THIS Publication, one of the beſt books on the elements of Botany, is unluckily the reſult of a literary robbery, being a copy of the lectures delivered by the celebrated *Desfontaines*, who has in different Journals re-claimed it, and has reprobated this abuſe of confidence, and this ſort of ſpoliation of the product of his ſtudies. However, though the author is juſtly to be blamed on this account, yet we cannot forbear to confeſs, that he has done much ſervice to young botaniſts, by putting into their hands theſe leſſons, of which they particularly ſtand in need, and which Desfontaines would probably not have ſo ſoon publiſhed, as he is only accuſtomed to bring before the public, the reſults of a long and laborious ſtudy. We recommend our readers, who are happy enough to find pleaſure and comfort in the ſtudy of Botany, to procure this work, which is a good compendium of that ſtudy.

---

*Illuſtratio Iconographica Inſectorum, quae in muſæis Pariſinis obſervavit et in lucem, edidit J. Chr. Fabricius, præmiſſis ejuſdem Deſcriptionibus; accedunt Species plurimae, vel minus, aut nondum cognitae; Authore Ant. Coquebert, Societ. Philomat. et Hiſtoriae Natur.* Paris, Socio, Tabular, Decas 1, Pariſiis, Typis Didot, natu majoris, An. 7, 4to. vellum paper, figures, coloured.

MR. FABRICIUS has deſcribed in his Syſtematic Entomology, and the Supplement to it, ſeveral new inſects, which he ſaw in the different different collections at Paris. His descriptions, however, being not always sufficient for determining the species he had in view, the great difficulties hence arising can only be removed by good figures of the orignals, signed by his hand; by which means these objects will, as it were, be multiplied and perpetuated, and which, if once destroyed, may perhaps not be found again. It is therefore incumbent upon us to do proper justice to the zeal and merit of Cit. Coquebert, author of the work we are here announcing, which consists in representing by figures, coloured after his own drawings, the insects observed in the Paris cabinets, and described by Mr. Fabricius; in adding the description and synonimous terms of that naturalist, and in publishing other new species, or those that are less known. The celebrated artists, Cit. Maleuvre for engraving, and Didot the elder for the typographical part, have assisted in this interesting work, and seconded the pencil of a man, who knows how necessary it is to observe and study Nature, before he attempts to copy her. The 10 first plates contain about 120 figures, a great number of which present the insects in detail. He begins with those that are particularly subject to be destroyed, belonging at the same time to less numerous classes than others, viz. the *Symitotes*, *Peezotes*, and *Ryngotes* of Fabricius. North America, Cayenne, the Barbary states, and the south of France, are the principal countries from which the species he has figured are brought. The new genera of Delphax, Oryssus, and Psocus are here figured, with an accurate representation of their organs of manducation, in which he has been much assisted by Cit. Latraille, to whom the plate is entirely owing of the little known genus Psocus, which on account of their minuteness and fugacity, had hitherto escaped the observation of naturalists. Some anatomical observations, new synonimous terms, and other remarks, give an additional value to this work; and we are convinced that it will meet with the most favourable reception by every naturalist and amateur.

---

### *Mr. Davy's Chemical and Philosophical Researches.*

[ Concluded from pp. 360—364, of our last. ]

" I was awakened by head-ache and painful nausea. The nausea continued even after the contents of the stomach had been ejected. The pain in the head every minute increased; I was neither feverish nor thirsty; my bodily and mental debility were excessive, and the pulse feeble and quick.

" In this state I breathed for near a minute and half five quarts of gas, which was brought to me by the operator for nitrous oxyde; but as it produced no sensations whatever, and apparently rather increased my debility, I am almost convinced that it was from some accident, either common air, or very impure nitrous oxyde.

" Immediately after this trial, I respired 12 quarts of oxygene for near four minutes. It produced no alteration in my sensations at at the time; but immediately after I imagined that I was a little exhilarated.

"The head-ache and debility ftill however continuing with violence, I examined fome nitrous oxyde which had been prepared in the morning, and finding it very pure, refpired feven quarts of it for two minutes and half.

"I was unconfcious of head-ache after the third infpiration; the ufual pleafurable thrilling was produced, voluntary power was deftroyed, and vivid ideas rapidly paffed through my mind; I made ftrides acrofs the room, and continued for fome minutes much exhilarated. Immediately after the exhilaration had difappeared, I felt a flight return of the head-ache; it was connected with tranfient naufea. After two minutes, when a fmall quantity of acidified wine had been thrown from the ftomach, both the naufea and head-ache difappeared; but languor and depreffion, not very different in degree from thofe exifting before the experiment, fucceeded. They however, gradually went off before bed time. I flept found the whole of the night except for a few minutes, during which I was kept awake by a trifling head-ache. In the morning, I had no longer any debility. No head-ache or giddinefs came on after I had arifen; and my appetite was very great.

"This experiment proved, that debility from intoxication was not increafed by excitement from nitrous oxyde. The head-ache and depreffion, it is probable, would have continued longer if it had not been adminiftered. Is it not likely that the flight naufea following the effects of the gas was produced by new excitability given to the ftomach?

"To afcertain with certainty, whether the moft extenfive action of nitrous oxyde compatible with life, was capable of producing debility, I refolved to breathe the gas for fuch a time and in fuch quantities, as to produce excitement equal in duration and fuperior in intenfity to that occafioned by high intoxication from opium or alcohol.

"To habituate myfelf to the excitement, and to carry it on gradually,

"On December 26th, I was inclofed in an air-tight breathing box, of the capacity of about 9 cubic feet and half, in the prefence of Dr. Kinglake.

"After I had taken a fituation in which I could by means of a curved thermometer inferted under the arm, and a ftop-watch, afcertain the alterations in my pulfe and animal heat, 20 quarts of nitrous oxyde were thrown into the box.

"For three minutes I experienced no alteration in my fenfations, though immediately after the introduction of the nitrous oxyde the fmell and tafte of it were very evident.

"In four minutes I began to feel a flight glow in the cheeks, and a generally diffufed warmth over the cheft, though the temperature of the box was not quite 50°. I had neglected to feel my pulfe before I went in; at this time it was 104 and hard, the animal heat was 98°. In ten minutes the animal heat was near 99°, in a quarter a quarter of an hour 99.5°, when the pulse was 102, and fuller than before.

"At this period twenty quarts more of nitrous oxyde were thrown into the box, and well-mingled with the mass of air by agitation.

"In twenty-five minutes the animal heat was 100°, pulse 124. In thirty minutes, twenty quarts more of gas were introduced.

"My sensations were now pleasant; I had a generally diffused warmth without the slightest moisture of the skin, a sense of exhilaration similar to that produced by a small dose of wine, and a disposition to muscular motion and to merriment.

"In three quarters of an hour the pulse was 104, and animal heat not 99,5°, the temperature of the chamber was 64°. The pleasurable feelings continued to increase, the pulse became fuller and slower, till in about an hour it was 88°, when the animal heat was 99°.

"Twenty quarts more of air were admitted. I had now a great disposition to laugh, luminous points seemed frequently to pass before my eyes, my hearing was certainly more acute, and I felt a pleasant lightness and power of exertion in my muscles. In a short time the symptoms became stationary; breathing was rather oppressed, and on account of the great desire of action, rest was painful.

"I now came out of the box, having been in precisely an hour and a quarter.

"The moment after, I began to respire twenty quarts of unmingled nitrous oxyde. A thrilling extending from the chest to the extremities was almost immediately produced. I felt a sense of tangible extension highly pleasurable in every limb; my visible impressions were dazzling and apparently magnified, I heard distinctly every sound in the room, and was perfectly aware of my situation. By degrees, as the pleasurable sensations increased, I lost all connection with external things; trains of vivid visible images rapidly passed through my mind, and were connected with words in such a manner, as to produce perceptions perfectly novel. I existed in a world of newly connected and newly modified ideas. I theorised; I imagined that I made discoveries. When I was awakened from this semi-delirious trance by Dr. Kinglake, who took the bag from my mouth, indignation and pride were the first feelings produced by the sight of the persons about me. My emotions were enthusiastic and sublime; and for a minute I walked round the room perfectly regardless of what was said to me. As I recovered my former state of mind, I felt an inclination to communicate the discoveries I had made during the experiment. I endeavoured to recall the ideas; they were feeble and indistinct; one collection of terms, however, presented itself: and with the most intense belief and prophetic manner, I exclaimed to Dr. Kinglake, "*Nothing exists but thoughts!—the universe is composed of impressions, ideas, pleasures, and pains!*"

"About three minutes and half only had elapsed during this experiment, experiment, though the time, as meafured by the relative vividnefs of the recollected ideas, appeared to me much longer.

"Not more than half of the nitrous oxyde was confumed. After a minute, before the thrilling of the extremities had difappeared, I breathed the remainder. Similar fenfations were again produced; I was quickly thrown into the pleafurable trance, and continued in it longer than before. For many minutes after the experiment, I experienced the thrilling in the extremities, the exhilaration continued nearly two hours. For a much longer time I experienced the mild enjoyment before defcribed connected with indolence; no depreffion or feeblenefs followed. I ate my dinner with great appetite, and found myfelf lively and difpofed to action immediately after. I paffed the evening in executing experiments. At night I found myfelf unufually cheerful and active; and the hours between eleven and two, were fpent in copying the foregoing detail from the common-place book, and in arranging the experiments. In bed I enjoyed profound repofe. When I awoke in the morning, it was with confcioufnefs of pleafurable exiftence, and this confcioufnefs, more or lefs, continued through the day."

---

*A fecond Effay on Burns, in which an Attempt is made to refute the Opinions of* Mr. Earle,* *and* Sir W. Farquhar, *lately advanced, on the fuppofed Benefit of the Application of Ice in fuch Accidents: With Cafes and Communications, confirming the Principles and Practice brought forward in a former Effay. Alfo Proofs, particularly addreffed to Surgeons of the Army and Navy, of the Utility of the ftimulating Plan in the Treatment of Injuries caufed by the Explofion of Gunpowder.* By Edward Kentish, author of the former Effay.

In this Effay Mr. Kentifh has brought forward a number of proofs in addition to thofe mentioned in his former, of the fuperiority of his mode of treating burns with ftimulants over the cold applications generally recommended. His vicinity to the coal mines, where accidents frequently happen from the explofion of inflammable air, gives him extenfive practice in this branch of furgery, and his experience and ingenuity claim the attention and examination of his brethren on this fubject. He judicioufly remarks, "that if men would in general confine their efforts to a few points, fcience would make a more rapid progrefs, though there might be lefs brilliancy attached to individuals. It is by a divifion of labor that perfection is to be attained in the fcientific, as well as in the mechanic arts."

In order to demonftrate the *principle* on which Mr. Kentifh directs the application of ftimulants to burns, we quote the following paffage: "I have in my former Effay mentioned at fome length the effects of different degrees of temperature upon the animal fyftem. It will there appear, that animals endowed with the property

* Now Sir James Earle.

perty of life, have a power of preserving their exiftence under a varied degree of temperature proportioned to the perfection of their organization; and if gradually applied, the extent of the fcale is greater. Man will exift from zero to two hundred and fixty degrees of Fahrenheit's thermometer; though rapid changes much within that range will deftroy him. Such is his glaffy effence, that it appears neceffary to treat him as artifts do that fubftance, viz. to anneal him, by allowing his temperature to be changed gradually. Such is in fome delicate conftitutions the narrow fcale of accommodating power in the fyftem, that a change which appears infenfible to perfons of a more healthy frame, produces fuch difturbance in the actions of abforption and fecretion, as to bring on complaints of the moft ferious nature. Delicate ladies who keep clofe within the houfe, in going from one room to another bring on that ftate of the fyftem known by the term of *catching cold.* This ftate is alfo very frequently induced by a too fudden and great application of the ftimulus of heat, after the torpor induced from lowering the temperature only a few degrees. The effect of a diminifhed quantity of heat is proved by the facts adduced by Mr. Hunter, of the rabbits' ears, (*vide First Effay, p.* 108.) to have ftopped all action in the part; and the effect of increafed heat applied to my own body (ibid, p. 111.) proves the increafed action induced by heat. I do not mean by this to deny the power of fire combining with, and decompofing any part of the fyftem to which it has been fo applied. I am well convinced I could burn my finger off over an Argand's lamp; but after this decompofition, I fhould not expect that ice would draw out the caloric, and reftore me my finger. When caloric combines with any fubftance, a chemical union takes place; and when this is done with the living animal fibre, it is at the expence of a deftruction of organization, which no human art can reftore in that individual fibre. Yet fuppofing a finger, or part of a finger, to be fo deftroyed, there muft have been increafed action previoufly to deftruction; and where deftruction ceafes, increafed action, from this greateft of all known ftimuli, will begin. Now as I think it will be granted that parts cannot be reftored, thofe which we have to take care of muft clafs under the head of parts with increafed action. If this be admitted, I believe I have formerly pointed out the beft known modes of treatment. What to me is one of the moft convincing proofs of the truth of the principles I wifh to be made known, is, that the *inverfe* of them is proved to be true by the experience of all countries fubject to a diminifhed temperature; and, reafoning upon the fame data, Mr. Earle's principle muft be erroneous: for inftance, if in a *plus* of heat it fhould be drawn off by cold, in a *minus* of heat, it fhould be added by the body which is the readieft to part with its heat. The following cafe will illuftrate thefe principles: In the beginning of this year (1800) there was a fevere ftorm, in which many fhips were loft upon the coaft of Northumberland, and a great number of men perifhed from the intenfity of the cold. Three were taken to a fmall inn in a fifhing town on the coaft, and

 as as one was very much *froſt-bitten,* with the beſt intentions in the world the good people of the inn prepared a hot bath for him, in which having kept him a ſufficient time, they put him to bed, and gave him ſome hot ale and brandy. The conſequence of this treatment was, that the torpid actions of the ſyſtem were ſo rapidly put into motion, that the vital power was exhauſted in forty-eight hours, and the man died. The others, who fortunately had not ſo much *falſe* care beſtowed upon them, recovered with difficulty; for although they did not uſe the bath, they had a ſhare of the other ſtimuli applied, ſufficient to produce pernicious effects. Now had Mr. Earle's principle been right, the man would have lived, as they took the readieſt way of applying, both externally and internally, the *deficient heat.* Had the oppoſite practice been taken with this man, I have very little doubt but he would have recovered: the froſt-bitten parts (the extremities, both ſuperior and inferior) ſhould have been thawed in water not above 40 degrees; he ſhould then have baen put into a cold bed in a room without a fire; and if he had been allowed any ſtimulating drink (ſuch as ale) it at leaſt ſhould have been cold: but of the propriety of allowing, at an *early* period, even this ſtimulus, of *cold fermented* liquor, I have my doubts. For the danger of allowing too great ſtimulus after what is termed an accumulation of irritability, I refer the reader to accounts of ſhip-wrecked people: the narrative of the loſs of the Bounty will ſupply them with ſufficient obſervations on this head."

The ingenious author purſues the *principle* of treatment recommended in his former Eſſay, though in the detail of practice he has been induced to make ſome alterations, which he thinks highly advantageous. "In the firſt ſpecies, *where the action of a part only is increaſed,* I have not found any thing better for the firſt application than the *heated* Ol. Terebinth. and the digeſtive thinned with the ſame. In ſuperficial burns, when the *pain* has ceaſed, it will be adviſeable to deſiſt from this application in about four and twenty hours, as that time in many caſes will be ſufficient, and at the ſecond dreſſing a digeſtive ſufficiently thinned with common oil, will be adequate to the caſe; and on the third day to begin with the ceratum e lapide calaminari. I have frequently ſeen ſecondary inflammation excited by the remedy, which in the firſt inſtance puzzled and perplexed me conſiderably: I have likewiſe been informed of this conſequence by ſeveral gentlemen. The moſt certain *remedy* for this unpleaſant ſymptom is to apply a plaſter with digeſtive thinned with oil, or a plaſter of cerate, and over that *a large warm* poultice. This moſt effectually takes off the irritation of the part, and the cerate will finiſh the cure. Should there be much uneaſineſs of the ſyſtem, an anodyne proportioned to the age of the patient ſhould be given."

Mr. K. is very ſevere upon Dr. Underwood in his chapter on burns and ſcalds (*vide Diſeaſes of Children, Vol. 2, p.* 106.) for preſcribing applications whoſe actions and effects are ſo diametrically oppoſite.

---

*A Treatiſe*

*A Treatise on the Bath Waters; by* George Smith Gibbes, M. D. F. R. S. &c. 8vo. p. 71. London, 1800, Robinsons.

As the subſtance of this ſcientific and correct work has already appeared in Mr. Nicholſon's Journal, and as Dr. Saunders has given the reſults of the analyſis in his work on mineral waters, of which we gave ſome account in our laſt Number, it will not be neceſſary for us to make long extracts.

" With ſuch powerful effects," the Author obſerves, " as the Bath Waters are known to produce on the human conſtitution, it is aſtoniſhing to find that ſo few active principles ſhould have been diſcovered by thoſe learned men who have made them the ſubject of their enquiries. It has been alleged, that all attempts to diſcover the properties or effects of mineral waters on the human body by chemical experiment or analyſis are vain and inſignificant, as many effects are produced by them not to be accounted for from any diſcoverable impregnation. The learned Dr. Falconer very juſtly combats this opinion. He obſerves, " that ſuch reaſoning, " if applied equally to all branches of learning, would preclude " all ſearch whatever; and, had it formerly obtained, would have " prevented our acquiſition of many uſeful and important diſco- " veries which we now enjoy." The Doctor, with that liberality which diſtinguiſhes the character of the man of learning, obſerves, that in diſcuſſions on ſubjects of ſcience, when opinions alone are controverted, it may be alleged, that no apology is neceſſary; as candour ſhould be the inſeparable attendant on learning; and no man of ſcience ſhould be aſhamed to receive information, from whatever channel it may be derived. As an apology for offering to the world the preſent performance, it ought to be recollected that the ſcience of chemiſtry has within theſe few years made very rapid advances, and the cultivators of it have now many more fixed principles, on which to found their reaſonings, than could have been ſuſpected by thoſe who applied to it but a few years ſince. Even the exiſtence of many very active ſubſtances was then totally unknown. Many agents and principles, which, a few years back, were believed to regulate the phenomena of Nature, are now proved to have had no exiſtence but in the imaginations of the cultivators of this ſcience. A ſcience which comprehends ſo large a field of inquiry muſt doubtleſs be ſubject continually to changes, according to the advances which are made in it. The detection of a new principle muſt, in every ſcience, alter the reaſonings reſpecting the phenomena which it preſents.

" There are daily poured forth from the ſprings at Bath, upwards of two thouſand hogſheads of water; which water is heated conſiderably above the temperature of ordinary water. As a matter of curioſity, I find that the quantity of heat which is evolved by them in the courſe of one year, above the medium heat of other ſprings, would render above ſeven hundred million of cubic inches of iron red hot.

" The following is a ſtatement of the heat and quantity of the Bath Waters:

The

| | Tuns. | Hhds. | Gal. |
|---|---|---|---|
| The King's Bath contains - - - - | 314 | 0 | 36 |
| The Queen's Bath - - - - - - - | 81 | 3 | 11 |
| The Crofs Bath - - - - - - - - | 53 | 0 | 47 |
| The Hot Bath - - - - - - - - | 54 | 0 | 27 |
| | 502 | 4 | 58 |

" Sixteen ounces or forty cubic inches of water at 55° of heat were raifed by a cubic inch of iron red hot to 90°.

" One cubic inch, of red hot iron, would raife fifteen cubic inches of water, at 55° of heat, to the heat of the Bath waters ; and there arife two thoufand hogfheads of water every day from the fprings.

" The temperature of the King's Bath, and Hot Bath, is about 114° of Fahrenheit; by pumping a confiderable time they have been found to raife the thermometer to 116°, and even to 118°. The Crofs Bath is of fomewhat a lower temperature, about 110° or 112°.

" It is difficult to afcertain with precifion the heat of the Bath waters. When examined under the fame circumftances, their temperature appears uniform. The regular temperature of the Bath waters, and the length of time that they have been known to pour forth their hot ftreams, would induce us to think that the caufe of their heat is uniform, and very deeply fituated in the bowels of the earth. I beg leave to offer a conjecture refpecting that regularity of their temperature; which is, that at fome great depth in the earth, they are at a very high temperature, and that in coming up to the furface of the earth, their temperature is lowered to the degree they are found to poffefs. Thus it fhould feem that they are analogous to the Geyzer in Iceland, and that at a certain depth, they would be found to have nearly the fame appearances. That they are not of the nature of common fprings of water is evident from their not being affected by the viciffitudes of weather, nor from the alternation of wet or dry feafons.

" The following extract, however, from Lord Gardenftone's travelling memorandums, feems to fhew that water does fometimes acquire a confiderable temperature at no great diftance from the furface of the earth.

" Aix in Provence."

" Dr. Philips informed me of a remarkable fact relative to thefe " waters. About three or four years ago, the inhabitants were " alarmed by a fudden and great defect in the ufual flow of water " from their fountains. The flow gradually diminifhed, and in a " few days they were almoft dry; happily the caufe of this fcar- " city was foon difcovered and eafily remedied. In fact, a farmer " about the diftance of half an Englifh mile from Aix, had, " at this time, on fome fcheme of improvement, dug up part of " his ground, when, at a fmall depth from the furface, a body of " water rufhed out and continued to flow very plentifully. The " fact being reported at Aix, they conjectured that the farmer had

" thus

" thus accidentally fallen upon and diverted the ſtream which " ſupplied their fountains; but, upon inquiry, the farmer's ſtream " was found to have no degree of heat; on the contrary, it was " a very cold ſpring water.

" The experiment however was made. The farmer's ſtream " was replaced, and immediately the fountains of Aix were re- " pleniſhed with the ſame plenty and quality of water as formerly. " Thus it appears, with certainty, that this water acquires its " heat in the courſe of running from the farmer's ground to Aix; " but how or where it is impregnated with the quality of heat, is " not yet determined."

" I could mention a great variety of inſtances where the Bath waters have produced great effects in diſeaſe; but as I intend to make the medical properties of theſe waters the ſubject of the ſecond part of this treatiſe, I muſt content myſelf at preſent with expreſſing a hope that even thoſe who have hitherto conſidered theſe waters as inefficacious, will be inclined to agree with me, in allowing that theſe principles muſt have great medical powers.

" According to my experiments I beg leave to make a ſtatement of the Bath waters in the following manner:

1. The temperature at medium in the King's Bath 114°, in the Hot Bath a little above that of the King's Bath, and in the Croſs Bath about 96°.

2. In the water carbonic acid gas and azotic gas in very ſmall quantities. The carbonic acid ſurſaturates the carbonate of lime which is evolved by boiling. The following aëriform fluids eſcape from the ſprings through the water, and appear in bubbles on the ſurface:

   *a.* Azotic gas - - - - ,80.
   *b.* Carbonic acid gas - - ,15.
   *c.* Oxygene gas - - - - ,05.

3. Iron in a ſtate of extreme diviſion, the quantities in conſequence of its apparent volatility not to be eſtimated. According ſo ſome writers, the King's Bath contains the largeſt portion.

4. Sulphate of lime, or ſelenite in the proportion of ,40 of the ſolid reſiduum.

5. Superſaturated carbonate of lime ,20.

6. Silex ,15.

7. Alum, or ſulphate of alumine, ,05.

8. Common ſalt and ſulphate of ſoda ,20.

" The ſolid matter forms about a 660th part of theſe waters.

" The ſand which is thrown up by theſe ſprings is compoſed of ſilex, ſelenite, carbonate of lime, ſome ſulphur, and ſome particles of iron which have been found to be attracted by the magnet."

---

*The Hospital Pupil, or an Essay intended to facilitate the Study of Medicine and Surgery; in four Letters.* By JAMES PARKINSON. 8vo. pp. 159, price 3s. 6d. London, 1800. Symonds, &c.

THIS induſtrious and benevolent writer is ſo well known to the medical world, that our commendation can add nothing to his fame.

fame. In country towns, from neceffity, and frequently in London, from choice, the fame perfon acts in the character of Surgeon, Apothecary, Accoucheur, and Phyfician. It is the education of fuch a perfon that Mr. P. has principally in view. The firft letter is on the qualifications and difpofitions neceffary for a youth intended for the profeffion of Medicine or Surgery. The fecond, on the beft method of educating a medical ftudent, with an improved courfe of Hofpital Studies.

" The grand object," Mr. P. obferves, " which next calls for confideration, is what is the beft fituation in which a youth can be placed; and what is the mode of ftudy, which will moft certainly fecure him all the theoretical and practical knowledge, in the fcience of healing, which, in the prefent ftate of the fcience, can be communicated.

" According to the prefent fyftem, the firft care of a parent, who has a fon whom he intends fhall be educated in both branches of the healing art, is to find out fome gentleman of refpectability who is properly eftablifhed as a furgeon and apothecary. With him, paying a proper premium, he fixes his fon, who has received a common fchool education, as an apprentice for feven years. At the end of this period he is, in general, fent to one of the hofpitals in the metropolis, where he attends the lectures, and witneffes the practice of the hofpital for a twelvemonth, or even lefs time: and then, if a favourable opportunity offers, takes charge of the health of fome populous neighbourhood.

" Now, on full conviction, I affert, that of all the modes which could be devifed for a medical and chirurgical education, this is the moft abfurd; and is the one which would moft certainly exclude a young man from the chance of acquiring that knowledge which the important fituation he is about to fill fo imperioufly demands.

" To put the fubject in a fair point of view, it will be proper firft to take a glimpfe of the manner in which the days of a young man, thus difpofed of, are fpent; and then a fair eftimation may taken of the advantages and difadvantages which belong to this mode of communicating inftruction.

" The firft four or five years are almoft entirely appropriated to the compounding of medicines; the art of which, with every habit of neceffary exactnefs, might be juft as well obtained in as many months. The remaining years of his apprenticefhip bring with them the acquifition of the art of bleeding, of dreffing a blifter, and, for the completion of the climax—of exhibiting an enema. If he be bleft with a mind fo alert, that even this fituation, fo ill calculated to call it into action, cannot render it torpid, he feizes every opportunity of gaining information; he perufes and re-perufes the Difpenfatory, ftudies fome obfolete practice of medicine, and dofes over fome treatife on phyfiology. Unknowing from actual infpection the form, ftructure, and fituation of the feveral parts of the human body, his ideas of anatomy cannot but be exceedingly incorrect: phyfiological facts will be hardly within his comprehenfion.

fion, and his ideas refpecting the treatment of difeafes muft be almoft pure y empircal.

"A young man, fuch as I have already defcribed, at the expiration of his apprenticefhip, or foon after, enters himfelf as a pupil of an hofpital; and as anxious, perhaps, to fulfil the period of his pupillage, as he was to accomplifh the term he has juft finifhed, he commences, at the fame time, to attend the feveral lectures, and to act as a dreffer. But when, as it muft often happen, from the mif-direction of his earlier ftudies, of which I have juft complained, he begins his courfe of obfervation, or of dreffing, with but a flight knowledge of anatomy, and of the firft principles of medicine and furgery, it muft be evident that he muft be incompetent to derive the full advantages which thefe opportunities offer to him.

"From what I have here, without exaggeration, ftated, do you not perceive that a young man, who is placed behind the counter of an apothecary, for feven years, and receives his hofpital education in the manner I have defcribed, muft be robbed of his fair chance of becoming a proficient in his profeffion; and be too likely to enter upon the exercife of it, without being in poffeffion of the degree of knowledge, neceffary for the due performance of the duties of fo important a ftation?

"From your defcribing your fon to be of an age fit for apprenticefhip, as I have already remarked, and from the remembrance retain of him, I fuppofe him to be about fourteen or fifteen years of age. The plan which I fhall propofe will, therefore, be fuch as I think may be commenced by a youth of that age.

"The knowledge of ANATOMY being neceffary, I am convinced, to be attained directly, he fhould, therefore, as foon as poffible, engage himfelf in a courfe of anatomical lectures. My friend, I know you well, and therefore, without any violent ftretch of imagination, can fancy that you, as well as many others who may read this paffage, will exclaim—what! attend a courfe of anatomical lectures at fourteen years of age! But a little attention will, I truft, evince the propriety of the recommendation.

"Let it be confidered that anatomy is the very alphabet of phyfiology, which teaches the knowledge of the animal œconomy; and that, by anatomy alone, we learn the natural ftate of parts, as well as the changes produced by difeafe, the removal of which will be fo much the object of his future endeavours. An early knowledge of anatomy muft, therefore, be obtained: but as three or four months muft now elapfe before any courfe of anatomical lectures commences, let the intermediate time be employed in rendering his knowledge of the *Latin* and *Greek* more perfect; but particularly of the former.

"He fhould, at this period alfo, read fome elementary treatife on anatomy; fuch as Dr. Hooper's Anatomical Pocket-book, fince he will thereby gain fome information refpecting thefe parts on the depicting of which his pencil is exercifed; at the fame time, that it will be to him as a nomenclature, teaching him the meaning of the various terms ufed in the fcience. He may alfo employ himfelf, at times, in obtaining fome general knowledge of the feveral branches of NATURAL PHILOSOPHY.

" By a fedulous application he will thus render himfelf capable not only of comprehending the language of a lecturer; but alfo of better accompanying the lecturer, in the defcription of the various parts of the human body, and of their feveral offices. A foon as the opportunity prefents, he fhould commence his attendance on anatomical lectures; but not without having impreffed on his mind, that his future progrefs in his ftudies depends on his prefent vigilance and attention. Thofe hours which his lectures do not engage fhould be employed in tracing over the parts which have been the fubject of the preceding lecture, in fome anatomical work, fuch as Winflow's Syftem of Anatomy; or, in fome accurately drawn plates, fuch as thofe of Albinus, Euftachius, &c. by occafionally copying of which he will now much accelerate the obtaining of anatomical knowledge. In this manner, with two courfes of lectures, he fhould pafs the winter.

" In the feafon which intervenes between this period and the next winter courfe of lectures, he may be employed in the reading of fome fyftem of anatomy, as well as of phyfiology, written in Latin, fuch as the *Prælectiones Anatomicæ* of Leber, and the *Primæ Lineæ Phyfiologiæ* of Haller; by doing this he will keep up, at the fame time, his anatomical knowledge and his acquaintance with the Latin.

" With his next courfes of anatomical lectures fhould be combined lectures on PHYSIOLOGY. Thefe, from the anatomical information he has now gained, and from the ftudies which have lately employed him, he will now be able to underftand, fufficiently to fecure his attention, and to excite that intereft which due to a fcience, the knowledge of which is of fo much confequence to him. During his attendance on the firft courfe of this fecond fet of anatomical lectures, he fhould, at the lateft, commence the exercife of PRACTICAL ANATOMY. If this be properly attended to, and a due portion of the hours, not employed in diffection and lectures, be given to revifing and copying the fhort-hand notes of the phyfiological lectures, his time will be fairly, and I doubt not fuccefsfully employed.

" Having thus completed his fecond feafon, his mind will have now become well furnifhed with fundamental knowledge; and his fummer courfe of ftudies may be of courfe widened. His reading will now be extended to the more voluminous works of Haller, Monro, &c. by which, with occafional reference to his notes, and the exercife of his pencil, his anatomical and phyfiological attainments will be confiderably increafed. He will now read fome compendium of modern CHEMISTRY, perhaps the one which I have publifhed for the ufe of ftudents may be as proper as moft, or fhould he choofe a larger work, the Principles of Chemiftry, by Dr. Gren, is recommendable.

" The other branches of natural philofophy muft now be again earneftly earneftly attended to; and, if poffible, the knowledge of them fhould be accelerated, by the happy illuftration which will be yielded, by a courfe of lectures and exhibition of experiments.

During this period he may alfo imprefs on his mind, fo ftrongly as to be prompt to his recollection, the generic characters, at leaft, of difeafe, from the Nofology of Cullen. After this he may read, with advantage, the Firft Lines of the Practice of Phyfic, by the fame celebrated writer. Some fyftematic treatife of furgery may now alfo engage his attention: and he fhould likewife obtain fome knowledge of the MATERIA MEDICA.

"He will now be prepared to refume his winter courfe of ftudies, which muft now comprife two courfes of lectures on CHEMISTRY, on the PRACTICE of PHYSIC, and on the PRACTICE OF SURGERY, and a continuance of anatomical lectures and diffections.

"His third term being thus filled up, and completed, he will be found qualified to attend to the demonftration of the nature of difeafe, and the effects of medicines, in CLINICAL LECTURES; he will now alfo be enabled to derive confiderable advantage by attending the hofpital as an attentive obferver."

The third letter contains directions for the profecution of hofpital ftudies, according to the *prefent* fyftem of medical education; and the laft fuggefts hints on entering into practice, and on medical jurifprudence, &c.

We earneftly recommend this work to all young men who are at prefent, or propofe to be, engaged in the ftudy of Medicine or Surgery.

---

*A Comparative Statement of Facts and Obfervations relative to the Cow-Pox, as publifhed by Doctors* JENNER *and* WOODVILLE. 4to. pp. 43. pr. 5s. London, 1800, Low, &c.

Dr. WOODVILLE'S fecond publication, entitled "*Obfervations on the Cow-Pox,*" were noticed at p. 256 of Vol. III. and we there intimated that they appeared to have been dictated by the fenforial powers of irritation and fenfation, rather than thofe of volition and affociation; and we then thought they required only to be left to the attempering effects of Time. This author, however, appears to think otherwife, and has attempted to affimilate thefe oils and acids by the *tertium,* reafon, or comparifon. The following extracts will enable our readers to judge of the author's candour and accuracy.

"To prevent the poffibility of mifreprefentation, Dr. Jenner's pofitions fhall be contrafted with correct extracts from Dr. Woodville's pamphlets.

"We fhall begin with the moft important pofition of Dr. Jenner:

'*That perfons, who have been affected with Cow-pox, are rendered 'perfectly fecure from the effects of variolous contagion.*'

* "It has been afferted, that perfons have had the Small-pox "after having been affected with the Cow-pox; and fome facts "have been publifhed with a view to fhow that inftances of this "kind have actually happened. But all thefe, as far as I have "feen, have been very defective in not affording fufficient proof, "that the affection fuppofed to have been the Cow-pox, was in "reality that difeafe. On the other hand, the inftances which "have been brought forward to prove that thofe who had under- "gone the genuine Cow-pox refifted the infection of the Small- "pox, are unqueftionably decifive, and fufficiently numerous to "eftablifh the fact in the moft fatisfactory manner. This circum- "ftance then appears to be as much a general law of the fyftem, "as that a perfon having had the Small pox is thereby rendered "unfufceptible of receiving the difeafe a fecond time. For of all "the patients whom I inoculated with variolous matter, after they "had paffed through the Cow-pox, amounting to upwards of 400, "none were affected with the Small-pox; and it may be remarked, "that nearly a fourth part of this number was fo flightly affected "with the Cow-pox, that it neither produced any perceptible in- "difpofition, nor puftules."

"The pofition of nearly equal import is,—*That the Cow-pox is* "*not contagious by effluvia.*"

† "One important advantage which the Cow-pox is fuppofed "to have over the Small-pox is, that the former is not a con- "tagious difeafe, and not to be propagated by the effluvia of per- "fons infected with it. This is certainly true when the diforder "is confined to the inoculated part, but where *it produces numerous* "*puftules upon the body*, the exhalation they fend forth is capable "of infecting others in the fame manner as the Small-pox. Two "inftances of cafual infection in this way have lately fallen under "my obfervation; in one the difeafe was fevere, and the eruption "confluent; in the other the difeafe was mild, and the puftules "few."

"The following pofition of Dr. Jenner next merits our attention; '*That no eruption, ending in variolous-like puftules, belongs to the* '*Cow-pox.*'

‡ "Although I differ in opinion from Dr. Jenner in not imput- "ing the puftular eruptions, produced in the cafes at the hofpital, "to any adulteration of the vaccine matter, employed in the "inoculations, yet I readily admit that *they have been*, and ftill "*continue to be, the effect* of fome *adventitious caufe, independent of* "*the Cow-pox*§."

'*That*

* See Dr. Woodville's Reports, page 154.

† See Dr. Woodville's Reports, page 153.

‡ See Dr. Woodville's Obfervations, page 18.

§ Dr. Woodville, "*in his Obfervations*," is at variance with the opinion he formerly advanced "*in his Reports*."—In the latter he afferts, that "*the Cow-pox* produces *numerous puftules upon the body*;" in the former, that the puftules "*have been*, and *continue to be, the effect* of fome *adventitious caufe in-* *dependent of the Cow-pox.*"

* '*That the Cow-pox is a much milder disease than the Small-pox,*' is Dr. Jenner's fourth position.

* "In regard to the comparative mildness of the vaccine and "variolous diseases, as produced from inoculation, I have been "enabled to give a very different report from that which I pub- "lished last year. The reason why several of the Cow-pox cases "then at the hospital proved severe, like those of the inoculated "Small-pox, has already been sufficiently explained, and will, I "trust, have the effect of placing the Cow-pock inoculation in a "more advantageous point of view than my former *Reports* pre- "sented."

The last position, and of least importance, is—"*That the Cow-pox proceeds from the diseased fluids of the horse.*

"In this Dairy Country, (says Dr. Jenner) a great number of "cows are kept, and the office of milking is performed indiscri- "minately by men and maid servants. One of the former having "been appointed to apply dressings to the heels of a horse affected "with *the grease*, and not paying due attention to cleanliness, in- "cautiously bears his part in milking the cows, with some parti- "cles of the infectious matter adhering to his fingers. When this "is the case, it commonly happens that a disease is communicated "to the cows, and from the cows to the dairy-maids, which "spreads through the farm until most of the cattle and domestics "feel its unpleasant consequences. This disease has obtained the "name of the *Cow-pox*."

Dr. Woodville, far from adopting this position, relates the following experiments, to prove that the disorder in question does not originate from any disease of the horse.

† "Conceiving that the distemper might be produced by ino- "culating the nipples of cows with the matter of the grease of "horses, in conformity with the opinion above stated, I proceed- "ed to try whether the Cow-pox could be actually excited in this "manner.

"Numerous experiments were accordingly made upon different "cows, with the matter of grease, taken in the various stages of "that disease, but without producing the desired effect.

"My friend, Mr. Coleman, the ingenious Professor at the Ve- "terinary College, likewise made similar trials, which proved "equally unsuccessful.

"Neither were inoculations with this matter, nor with several "other morbid secretions in the horse, productive of any effects "upon the human subject."

"Mr. Tanner being known to Dr. Woodville, and respected by him for his integrity, Dr. Woodville can justly appreciate the degree of credit that ought to be given to the following letter:

"Sir,

* See Dr. Woodville's Observations, p. 28.

† See Dr. Woodville's Reports, p. 6, 7, 8.

"Sir,

"Some Cow-pox matter on a thread was applied to the teat of "a cow on the part from which a fcab had been removed. I pro- "cured it from Mr. Fewfter, of Thornbury, who told me it had "been kept a long time, and that he did not think it poffible for "it to produce any effect. I went to the cow and examined the "part where it had been applied in five days after, but it had not "produced the fmalleft effect. Some limpid matter, juft taken "from the heel of a horfe, was then applied on the part, and on "the ninth day, when I firft examined it, I found that it had pro- "duced a complete vaccine puftule. From handling the cows teats "I became infected myfelf, and had two puftules on my hand, "which brought on inflammation, and made me unwell for feveral "days. The matter from the cow, and that from my own hand, "proved efficacious in infecting both human fubjects and cattle.

"I am, your's, &c.

"Thomas Tanner, V. S."

---

